# Environmental Bright Light Exposure, Depression Symptoms, and Sleep Regularity

**DOI:** 10.1001/jamanetworkopen.2024.22810

**Published:** 2024-07-17

**Authors:** Danielle A. Wallace, Susan Redline, Tamar Sofer, Joe Kossowsky

**Affiliations:** 1 Division of Sleep Medicine, Harvard Medical School, Boston, Massachusetts; 2 Division of Sleep and Circadian Disorders, Departments of Medicine and Neurology, Brigham and Women’s Hospital, Boston, Massachusetts; 3 Cardiovascular Institute, Beth Israel Deaconess Medical Center, Boston, Massachusetts; 4 Department of Epidemiology, Harvard T. H. Chan School of Public Health, Boston, Massachusetts; 5 Department of Biostatistics, Harvard T. H. Chan School of Public Health, Boston, Massachusetts; 6 Department of Anesthesia, Harvard Medical School, Boston, Massachusetts; 7 Department of Anesthesiology, Critical Care and Pain Medicine, Boston Children’s Hospital, Boston, Massachusetts

## Abstract

This cross-sectional study examines the associations between bright light therapy, sleep regularity, and depression symptoms among adults in the US.

## Introduction

Bright light therapy (BLT) may treat depression symptoms,^1^ but how light exerts mood-boosting effects is still under investigation. Here, we evaluate sleep regularity in the association between bright light and depression symptoms.

## Methods

This cross-sectional study of 2011-2014 National Health and Nutrition Examination Survey (NHANES) data, representative of the noninstitutionalized US population, included nonpregnant participants 18 years or older with valid light and actigraphy (wrist-worn GT3X+; ActiGraph) for bright light (time above lux threshold [TALT_1000_]) and sleep regularity index (SRI)^2^ measures (eMethods in Supplement 1) and follows STROBE guidelines. Depression symptoms were measured with the Patient Health Questionnaire-9 (PHQ-9; scores range from 0 to 30, with higher scores indicating greater frequency of depression symptoms). This analysis used publicly available, deidentified data from NHANES, which was approved by the Ethics Review Board of the Centers for Disease Control and Prevention. All participants provided written informed consent.

This analysis was conducted between March 2023 and May 2024. Associations between TALT_1000_, SRI, and PHQ-9 score were tested in weighted linear, quasi-Poisson, and logistic regression models adjusted for age, sex, race and ethnicity, season, employment, physical activity, sleep duration, body mass index (BMI), and serum cotinine (eMethods in Supplement 1). Sex differences were tested with weighted *t* tests. In exploratory analyses, associations between TALT_1000_ and vitamin D, vitamin D and SRI, and TALT_1000_ and SRI with vitamin D as a potential mediator were tested in weighted linear regression models. *P* < .05 was considered statistically significant. Analyses were performed in R, version 4.1.1 (R Foundation).

## Results

There were 6639 participants included in the analysis (mean [SD] age, 49.41 [17.41] years; 52.2% female; self-reported 8.3% Mexican American, 4.4% non-Hispanic Asian, 11.3% non-Hispanic Black, 67.7% non-Hispanic White, 5.7% other Hispanic, and 2.6% other or multiracial). Male participants had greater TALT_1000_ and fewer depression symptoms than female participants (Table 1). Greater TALT_1000_ was associated with lower depression symptoms (β = −0.19; 95% CI, −0.34 to −0.04) but not more severe depression. TALT_1000_ was no longer associated with depression score after adjusting for SRI (β = −0.11; 95% CI, −0.26 to 0.04; *P* = .13). Longer duration in bright light was associated with more regular sleep (β = 1.60; 95% CI, 0.95-2.25). Likewise, more regular sleep was associated with a lower depression score (β = −0.05; 95% CI, −0.06 to −0.03) and lower odds of mild or more severe depression (OR = 0.98; 95% CI, 0.97-0.99) (Table 2). In exploratory analyses, more vitamin D was associated with greater TALT_1000_ and more regular sleep but not depression symptoms.

**Table 1.** Variable Mean Values by Total Sample and by Sex^a^

Variable	All (n = 6639)	No. missing	Male (n = 3208)	Female (n = 3431)	*P* value^b^
Age, mean (SD), y	49.41 (17.41)	0	48.52 (17.37)	50.22 (17.41)	<.001
Sex, No. (%)					
Female	3431 (52.2)	0	NA	NA	NA
Male	3208 (47.8)				
Race and ethnicity, No. (%)					
Mexican American	797 (8.3)	0	389 (8.9)	408 (7.7)	.03
Non-Hispanic Asian	720 (4.4)		344 (4.2)	376 (4.6)	
Non-Hispanic Black	1600 (11.3)		783 (10.7)	817 (11.9)	
Non-Hispanic White	2707 (67.7)		1327 (68.1)	1380 (67.4)	
Other Hispanic	626 (5.7)		267 (5.2)	359 (6.1)	
Other^c^ or multiracial	189 (2.6)		98 (3.0)	91 (2.3)	
BMI, mean (SD)	29.16 (6.97)	70	28.86 (6.05)	29.42 (7.71)	.02
Serum cotinine, mean (SD), ng/mL	50.80 (121.99)	314	61.04 (137.65)	41.36 (104.65)	<.001
Season, May 1 to October 31, No. (%)	3401 (54.4)	0	1608 (53.9)	1793 (54.9)	.42
Employment status, yes, No. (%)	3352 (57.9)	2	1773 (64.9)	1579 (51.5)	<.001
Daily Monitor–Independent Movement Summary physical activity value, mean (SD)	13 246 (3787)	0	12 863 (3839)	13 596 (3705)	<.001
Sleep duration, mean (SD), h	7.90 (1.52)	0	7.84 (1.60)	7.96 (1.44)	.005
TALT_1000_, mean (SD), h	1.12 (1.03)	0	1.39 (1.20)	0.87 (0.77)	<.001
SRI, mean (SD)	58.32 (15.96)	0	57.51 (16.25)	59.05 (15.66)	.009
Vitamin D, nmol/L, mean (SD)	71.60 (29.11)	282	67.67 (24.23)	75.22 (32.55)	<.001
PHQ-9, mean (SD)	3.31 (4.62)	438	2.74 (4.19)	3.84 (4.93)	<.001
PHQ-9 score ≥5, No. (%)	1638 (25.0)	438	647 (20.4)	991 (29.3)	<.001
PHQ-9 score ≥10, No. (%)	675 (9.8)	438	250 (7.4)	425 (12.1)	<.001

Abbreviations: BMI, body mass index (calculated as weight in kilograms divided by height in meters squared); NA, not applicable; PHQ-9, Patient Health Questionnaire-9; SRI, sleep regularity index; TALT_1000_, time above lux threshold.

SI conversion factor: To convert cotinine to nmol/L, multiply by 5.675.

^a^ Sample-weighted values are provided for mean (SD) and number (%), in addition to number of participants for categorical variables.

^b^ Sex differences were tested with 2-sample *t* tests or χ^2^ tests.

^c^ Cannot be specified further.

**Table 2.** Regression Model Associations Between Variables and Outcomes of National Health and Nutrition Examination Survey 2011-2014 Participants Aged 18 Years or Older (N = 6639)

Regression type	Variable	Outcome	Estimate (95% CI)^a^				*P* value			
			Crude	Model 1	Model 2	Model 3	Crude	Model 1	Model 2	Model 3
Linear regression	TALT_1000_, h	PHQ-9 score	−0.386 (−0.533 to −0.240)	−0.303 (−0.473 to −0.133)	−0.271 (−0.432 to −0.111)	−0.190 (−0.344 to −0.036)	<.001	.001	.002	.02
Quasi-Poisson regression	TALT_1000_, h	PHQ-9 score	−0.131 (−0.185 to −0.077)	−0.107 (−0.169 to −0.045)	−0.100 (−0.158 to −0.042)	−0.068 (−0.126 to −0.009)	<.001	.002	.002	.03
Logistic regression	TALT_1000_, h	PHQ-9 score ≥5	0.821 (0.751 to 0.898)	0.846 (0.767 to 0.933)	0.853 (0.768 to 0.947)	0.888 (0.792 to 0.995)	<.001	.002	.005	.04
Logistic regression	TALT_1000_, h	PHQ-9 score ≥10	0.842 (0.753 to 0.942)	0.869 (0.759 to 0.995)	0.880 (0.778 to 0.995)	0.948 (0.830 to 1.081)	.004	.04	.04	.39
Linear regression	TALT_1000_, h	SRI	2.947 (2.412 to 3.482)	2.996 (2.354 to 3.637)	2.906 (2.220 to 3.591)	1.604 (0.954 to 2.253)	<.001	<.001	<.001	<.001
Linear regression	SRI, 1-unit increase	PHQ-9 score	−0.065 (−0.078 to −0.053)	−0.070 (−0.084 to −0.057)	−0.060 (−0.073 to −0.048)	−0.047 (−0.062 to −0.032)	<.001	<.001	<.001	<.001
Quasi-Poisson regression	SRI, 1-unit increase	PHQ-9 score	−0.018 (−0.021 to −0.015)	−0.019 (−0.022 to −0.016)	−0.016 (−0.019 to −0.013)	−0.013 (−0.017 to −0.009)	<.001	<.001	<.001	<.001
Logistic regression	SRI, 1-unit increase	PHQ-9 score ≥5	0.972 (0.967 to 0.977)	0.969 (0.963 to 0.975)	0.973 (0.967 to 0.978)	0.978 (0.970 to 0.985)	<.001	<.001	<.001	<.001
Logistic regression	SRI, 1-unit increase	PHQ-9 score ≥10	0.970 (0.963 to 0.976)	0.966 (0.960 to 0.973)	0.972 (0.965 to 0.979)	0.978 (0.969 to 0.986)	<.001	<.001	<.001	<.001
Linear regression	TALT_1000_, h	Vitamin D	3.092 (1.936 to 4.248)	3.712 (2.625 to 4.799)	3.093 (1.948 to 4.237)	2.849 (1.747 to 3.951)	<.001	<.001	<.001	<.001
Linear regression	Vitamin D	SRI	0.070 (0.046 to 0.094)	0.078 (0.050 to 0.105)	0.073 (0.047 to 0.099)	0.046 (0.025 to 0.068)	<.001	<.001	<.001	<.001
Linear regression	Vitamin D	PHQ-9 score	−0.008 (−0.014 to −0.001)	−0.011 (−0.018 to −0.003)	−0.009 (−0.016 to −0.003)	−0.004 (−0.010 to 0.002)	.03	.009	.01	.20
Quasi-Poisson regression	Vitamin D	PHQ-9 score	−0.002 (−0.005 to −0.0002)	−0.003 (−0.006 to −0.001)	−0.003 (−0.005 to −0.001)	−0.001 (−0.003 to 0.001)	.03	.01	.01	.22

Abbreviations: PHQ-9, Patient Health Questionnaire-9; SRI, sleep regularity index; TALT_1000_, time above lux threshold.

^a^ Model 1 adjusted for age (years, continuous with natural spline *df* = 6), sex, and race and ethnicity. Model 2 adjusted for all the covariates included in model 1 in addition to season and employment status. Model 3 adjusted for all the covariates included in model 2 in addition to physical activity, mean sleep duration, body mass index, and blood cotinine level.

## Discussion

In this study, greater sleep regularity partly explained the association between greater bright light exposure and lower depression symptoms. More vitamin D was associated with more regular sleep timing but not depression. Our findings align with prior population-based research, supporting reduced depression among those with greater daytime light exposure.^3^ Bright light therapy can be effective at improving numerous mood outcomes,^4^ possibly through effects on sleep and the circadian system.^5^ Sleep regularity may be an important modifier of BLT^2^ and relate to chronotype and phase angle of entrainment.^6^ However, sleep regularity is not often considered in BLT. The phase-shifting effect of light may be limited in people with irregular sleep schedules, requiring a larger dose or altered timing of light exposure. Irregular sleepers may benefit from a light intervention.^2^

This study has strengths and limitations. It used a nationally representative sample of adult participants with objective measures of individual light exposure, actigraphy, and vitamin D. This is a cross-sectional analysis, so causality cannot be ascertained, and associations may be bidirectional. Although we are unable to rule out that mood may also influence time spent outdoors and/or bright light avoidance, our results support the need for further prospective analyses to test the causality of these factors. Future studies of BLT should consider the role of sleep regularity.

## Notes

Supplement 1.
eMethods.

eReferences

Supplement 2.
Data Sharing Statement
