# Supplementary material for: Environmental Bright Light Exposure, Depression Symptoms, and Sleep Regularity
Source: JAMA Netw Open. 2024 Jul 17;7(7):e2422810. doi: 10.1001/jamanetworkopen.2024.22810 (PMC11255914; doi:10.1001/jamanetworkopen.2024.22810)
Supplement: Supplement 1. — eMethods. eReferences [file jamanetwopen-e2422810-s001.pdf]

Supplemental Online Content

Wallace DA, Redline S, Sofer T, Kossowsky J. Environmental bright light exposure, depression symptoms, and sleep regularity. *JAMA Netw Open*. 2024;7(7):e2422810. doi:10.1001/jamanetworkopen.2024.22810

eMethods.

eReferences

This supplemental material has been provided by the authors to give readers additional information about their work.

© 2024 Wallace DA et al. *JAMA Network Open.*

eMethods.

Study population information

All data used in this analysis are publicly available from the NHANES website. In the NHANES study, demographic and lifestyle information was collected via survey questionnaires and interviews. Blood and physical measures were collected during physical examination in the Mobile Examination Center (MEC). Questionnaire and physical exam data collected during the 2011-2012 and 2013-2014 NHANES cycles were used in this analysis. This analysis used the “RIAGENDR” NHANES variable as a measure of sex. This variable was collected by either (a) asking the participant to self-report “male” or “female”, or (b) interviewer assessment. We acknowledge that this measure may not accurately capture sex (or gender); however, we use the term “sex” to maintain consistency throughout the manuscript. Race and ethnicity information was collected by asking the participant to self-report their belonging to predefined categories; participant response data was then coded by NHANES as “Mexican American”, “Non-Hispanic Asian”, “Non-Hispanic Black”, “Non-Hispanic White”, “Other Hispanic”, or “Other Race – Including Multi-Racial”. Self-reported race/ethnicity was included as a covariate to attempt to adjust for the influence of racism and bias on sleep health and the built environment.

Actigraphy measurement

NHANES collected activity and light data from wrist-worn GT3X+ ActiGraph devices (ActiGraph, Pensacola, FL) across 9 days of measurement following the physical exam during the 2011-2012 and 2013-2014 cycles. The GT3X+ has a light sensor which measures white light illuminance in lux for every epoch that activity is measured; the range of light values were capped at 2,500 lux in the NHANES data (for a range of 0-2,500 lux).

Actigraphy pre-processing methods

Minute-epoch actigraphy data was downloaded from the NHANES website and, for each participant, data was processed to include the first 7 valid days (from 12AM, 00:00, of day 2 onwards) of measurement. We utilized the Monitor Independent Movement Summary (MIMS) triaxial minute values as a measure of activity and the white light lux values as a measure of light. To additionally check and filter data quality, we internally created three quality control flags based on NHANES-provided quality control metrics: (1) if PAXFLGSM variable had a letter value; (2) if device was predicted to be off-wrist with PAXPREDM=3[1] or unknown with PAXPRED=4; and (3) if the triaxial activity value was <0. If any 1-minute sampling epoch contained one or more of these flags, the activity and light data for the epoch were recoded to missing (NA). A day was considered invalid if there was more than 6 hours of missing data, or if the sum of activity counts was <200. Participants with <6 valid days of measurement, who were <18 years old, and who reported being pregnant at the time of the exam were excluded. One participant with impossible $TALT_{1000}$ values was also excluded.

Derivation of bright light ($TALT_{1000}$) exposure, sleep regularity index (SRI), and average physical activity measures from actigraphy and light data

Following data cleaning of the NHANES-provided sleep-wake variable (as described above), binary values were used to calculate the SRI as previously described[2]. Briefly, the SRI is a measure that reflects the probability of being asleep or awake at any 1 point in time 24-hours apart[2]. We calculated the SRI for each participant using the NHANES sleep-wake variable, where PAXPREDM=1 was coded as awake (=1) and PAXPREDM=2 was coded as asleep (=0); instances where PAXPREDM=3 (non-wear) or PAXPREDM=4 (unknown) were coded to missing (NA). In instances with 10 or fewer consecutive missing epochs, NA value was imputed with the preceding value. Duration of time spent in bright light was calculated as the daily amount of time spent at or above 1,000 lux ($TALT_{1000}$) and then averaged across valid days. Average physical activity was calculated as the MIMS daily activity value (summed triaxial MIMS values, midnight-midnight), averaged across days. Sleep duration was calculated as the daily (midnight-midnight) number of minutes scored as sleep, averaged across days.

© 2024 Wallace DA et al. *JAMA Network Open.*

Vitamin D measurement
Serum samples were obtained during the physical exam and analyzed for 25-hydroxyvitamin D2 and 25-hydroxyvitamin D3 with ultra-high performance liquid chromatography-tandem mass spectrometry. Further methodology description can be found in the online NHANES documentation (2011-2012 data: https://wwwn.cdc.gov/Nchs/Nhanes/2011-2012/VID_G.htm; 2013-2014 data: https://wwwn.cdc.gov/Nchs/Nhanes/2013-2014/VID_H.htm). The summed concentration of D2 and D3 (nmol/L, continuous) was modeled.

Measurement of depression symptomology
Depression symptomology with DSM-IV criteria was measured using the Patient Health Questionnaire-9 (PHQ-9)[3], a 9-item questionnaire that evaluates the frequency of depression symptoms over the past 2 weeks. A cumulative summary score was calculated across the 9 items, with higher score indicating greater depression symptoms. Summary scores were also dichotomized using a threshold of ≥5 to indicate some depressive symptoms and a threshold of ≥10 to indicate possible major depressive disorder (MDD)[4].

Cotinine values below limit of detection (LOD)
Cotinine values below the limit of detection (LOD) were imputed by dividing the LOD by the square root of 2[5]. Age (years) was modeled as a continuous variable with natural splines and internal knots at sextiles (df=6).

Use of NHANES sample weights
Data were analyzed with 4-year (2011-2012, 2013-2014) combined MEC sample weights using the "survey" package[6] (v4.1-1) to derive population-based estimates. The use of NHANES sample weights is recommended to account for the complex survey design and obtain correct estimates that are representative of the population. More information regarding NHANES sample weights can be found here: https://wwwn.cdc.gov/nchs/nhanes/tutorials/weighting.aspx.

NHANES variables used in the analysis:

NHANES Variable Name	Variable Description
RIAGENDR	Sex
RIDRETH3	Race/ethnicity category
BMXBMI	Body mass index
RIDEXMON	Season of measurement
LBXCOT	Cotinine, measure in serum
LBDCOTLC	Cotinine comment whether below LOD or not
PAXLXSM	White light exposure in lux
PAXMTSM	Actigraphy triaxial activity MIMS minute values
PAXFLGSM	NHANES actigraphy quality flag
PAXPREDM	Actigraphy coding as awake, asleep, non-wear, or unknown
LBXVIDMS	Vitamin D2 and D3 (nmol/L) concentration, measured in serum
OCD150	Job status, coded as "yes" (ref) if response=1 ("working at a job or business") or =2 ("with a job or business but not at work"); otherwise "no"
RIDEXPRG	Pregnancy status; if response=1, considered pregnant and excluded from analysis

© 2024 Wallace DA et al. *JAMA Network Open.*

DPQ010 – DPQ100	Individual PHQ-9 questionnaire questions and responses; if value >3, coded as NA

eReferences:

1. John, D., Tang, Q., Albinali, F., and Intille, S. (2019). An Open-Source Monitor-Independent Movement Summary for Accelerometer Data Processing. Journal for the Measurement of Physical Behaviour *2*, 268–281. 10.1123/jmpb.2018-0068.

2. Phillips, A.J.K., Clerx, W.M., O'Brien, C.S., Sano, A., Barger, L.K., Picard, R.W., Lockley, S.W., Klerman, E.B., and Czeisler, C.A. (2017). Irregular sleep/wake patterns are associated with poorer academic performance and delayed circadian and sleep/wake timing. Sci. Rep. *7*, 3216. 10.1038/s41598-017-03171-4.

3. Spitzer, R.L., Kroenke, K., and Williams, J.B. (1999). Validation and utility of a self-report version of PRIME-MD: the PHQ primary care study. Primary Care Evaluation of Mental Disorders. Patient Health Questionnaire. JAMA *282*, 1737–1744. 10.1001/jama.282.18.1737.

4. Levis, B., Benedetti, A., Thombs, B.D., and DEPRESsion Screening Data (DEPRESSD) Collaboration (2019). Accuracy of Patient Health Questionnaire-9 (PHQ-9) for screening to detect major depression: individual participant data meta-analysis. BMJ *365*, l1476. 10.1136/bmj.l1476.

5. Hornung, R.W., and Reed, L.D. (1990). Estimation of average concentration in the presence of nondetectable values. Appl. Occup. Environ. Hyg. *5*, 46–51. 10.1080/1047322X.1990.10389587.

6. Lumley, T. (2004). Analysis of complex survey samples. J. Stat. Softw. *9*. 10.18637/jss.v009.i08.

© 2024 Wallace DA et al. *JAMA Network Open.*
