# Supplementary material for: Environmental Bright Light Exposure, Depression Symptoms, and Sleep Regularity
Source: JAMA Netw Open. 2024 Jul 17;7(7):e2422810. doi: 10.1001/jamanetworkopen.2024.22810 (PMC11255914; doi:10.1001/jamanetworkopen.2024.22810)
Supplement: Supplement 2. — Data Sharing Statement [file jamanetwopen-e2422810-s002.pdf]

Data Sharing Statement

Wallace. Environmental Bright Light Exposure, Depression Symptoms, and Sleep Regularity. *JAMA Netw Open*. Published July 17, 2024. doi:10.1001/jamanetworkopen.2024.22810

Data
Data available: Yes
Data types: Other (please specify)
Additional Information: All data used in this study is available through CDC NHANES
How to access data: The dataset and information regarding study design, measurement, and variables used in this secondary analysis are publicly available online from the CDC's NHANES website: https://www.cdc.gov/nchs/nhanes/index.htm.
When available: With publication

Supporting Documents
Document types: Other (please specify)
Additional Information: The dataset and information regarding study design, measurement, and variables used in this secondary analysis are publicly available online from the CDC's NHANES website: https://www.cdc.gov/nchs/nhanes/index.htm.
How to access documents: The dataset and information regarding study design, measurement, and variables used in this secondary analysis are publicly available online from the CDC's NHANES website: https://www.cdc.gov/nchs/nhanes/index.htm.
When available: With publication

Additional Information
Who can access the data: Data is available to anyone
Types of analyses: Any purpose
Mechanisms of data availability: Data are publicly available online from the CDC's NHANES website: https://www.cdc.gov/nchs/nhanes/index.htm.
